# A surprising complete response to cadonilimab in a primary metastatic cervical cancer: a case report

**DOI:** 10.3389/fimmu.2024.1494138

**Published:** 2024-11-26

**Authors:** Haijuan Yu, Jie Lin, Jian Chen, Lijun Chen, Jianping Zou, Bin Liu, Dan Hu, Youping Xiao, Linhao Yu, Yang Sun

**Affiliations:** ^1^ Department of Gynecology, Clinical Oncology School of Fujian Medical University, Fujian Cancer Hospital, Fuzhou, Fujian, China; ^2^ Department of Pathology, Clinical Oncology School of Fujian Medical University, Fujian Cancer Hospital, Fuzhou, Fujian, China; ^3^ Department of Radiology, Clinical Oncology School of Fujian Medical University, Fujian Cancer Hospital, Fuzhou, Fujian, China

**Keywords:** cadonilimab, recurrent and/or metastatic cervical cancer, complete response, HER-2, case report

## Abstract

The outcome of patients with recurrent/metastatic cervical cancer (R/M CC) is poor, with a 5-year survival rate of only 10%–20%. Recent advances in immunotherapy renewed its interest in R/M CC treatment. It has been suggested that cadonilimab, a novel bispecific antibody targeting programmed death 1 (PD-1) and cytotoxic T-lymphocyte antigen-4 (CTLA-4), significantly improved the survival outcomes of the R/M CC. In the present study, we reported a programmed death ligand 1 (PD-L1) and human epidermal growth factor receptor 2 (HER-2) positive CC case at stage IV who was treated with cadonilimab and achieved a surprising radiographic complete response (CR) for 10 months, even in the PD-L1 negative metastatic site. Demographic, clinical, histopathological, laboratory, treatment regime and imaging data were recorded. Unfortunately, the patient progressed rapidly during maintenance therapy when cadonilimab was replaced by sintilimab, the monoclonal antibody against PD-1, indicating the more powerful anti-tumor activity of dual blockade immunotherapy. To conclude, cadonilimab offers a promising and effective therapeutic approach for R/M CC. Notably, HER-2 is also expected to be a new reference target for cadonilimab therapy.

## Introduction

Cervical cancer (CC) is one of the most common cancers in the female reproductive system worldwide (1). with an estimated 661,021 new cases and 348,189 deaths in 2022. China accounts for 22.8% of the worldwide incidence and 16.0% of CC-related mortality (2). Treatment approaches and outcomes for CC patients are highly dependent on the disease stage at diagnosis. The five-year survival rate of patients with early-stage CC is above 90%. However, it dramatically drops to less than 20% in recurrent or metastatic CC (R/M CC) (3, 4), resulting in the median overall survival (OS) of 16.8 months (5–7). Therefore, new therapeutic options for R/M CC patients are desperately needed in the first as well as later lines (8, 9).

Immunotherapy has become a novel treatment option for patients with R/M CC. In the past decade, multiple clinical trials investigated the efficacy of immune checkpoint inhibitors (ICIs) (10), such as pembrolizumab, balstilimab, and nivolumab with objective response rates (ORRs) of 12%∼26% in second line-treatment for R/M CC (11–13). Keynote-826 trial showed pembrolizumab and chemotherapy with or without bevacizumab, as first-line therapy, resulted in significantly longer progression-free survival (PFS) and OS for the programmed death ligand 1 (PD-L1) combined positive score (CPS) ≥1 R/M CC (14, 15). However, the benefits were limited for patients with PD-L1-negative (16). The dual blockade immunotherapy to improve the efficacy of programmed death 1 (PD-1) monotherapy has been widely investigated (17, 18). For example, SHR-1701, a bifunctional antibody composed of the anti-PD-L1 agent and extracellular domain of the transforming growth factor-beta II (TGF-βII) receptor, only achieved an ORR of 15.6% in R/M CC patients based on NCT05179239 trial (19). However, dual blockade immunotherapy has been limited by severe toxicities (20).

Faced with such a dilemma, cadonilimab, a first-in-class bi-specific antibody targeting PD-1 and cytotoxic T lymphocyte antigen 4 (CTLA-4), has emerged to improve the prognosis of patients with R/M CC. Cadonilimab with crystalline fragments not only enhances the antitumor activity but also mutates to eliminate the Fc receptor and complement-mediated cytotoxic effects (21). Results from AK104-201 showed an inspiring ORR of 31.3% (31/99) [ORR _PD-L1 positive_ = 43.8% (28/64); ORR _PD-L1 negative_ = 16.7% (3/18)] (22) in the R/M CC who had failed previous platinum-containing chemotherapy with a low incidence of grade ≥3 immune-related adverse events (irAEs). Based on these results, cadonilimab was approved by China’s National Medical Products Administration for R/M CC as a second-line treatment in June 2022 (23).

Historically, disseminated cancers had a lower probability of a complete response (CR) even with high-intensity comprehensive treatments. However, combining immunotherapy and other comprehensive treatments could bring new hopes and clinical benefits to late-stage and metastatic disease. Our study presented a metastatic CC patient who obtained CR for 10 months with cadonilimab treatment. Furthermore, the toxicities associated with this salvage treatment were tolerable. This case aims to serve as a reference for treating R/M CC patients with similar presentations. Additionally, studies investigating the mechanisms underlying the promising therapeutic regimens need to be conducted in greater depth in the near future.

## Case presentation

**Figure 1:**
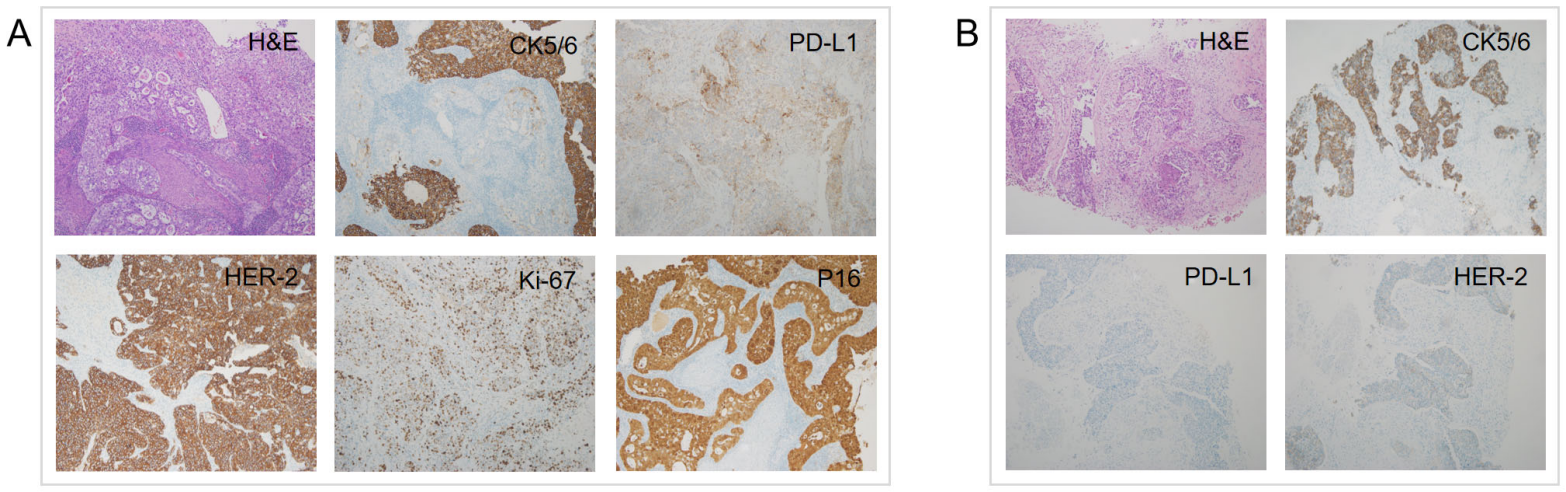
H&E and IHC of the patient’s cervix **(A)** and left supraclavicular lymph node **(B)** biopsy pathology (H&E staining, ×100; IHC, ×100). H&E, Hematoxylin and eosin stain; IHC, immunohistochemistry.

**Figure 2:**
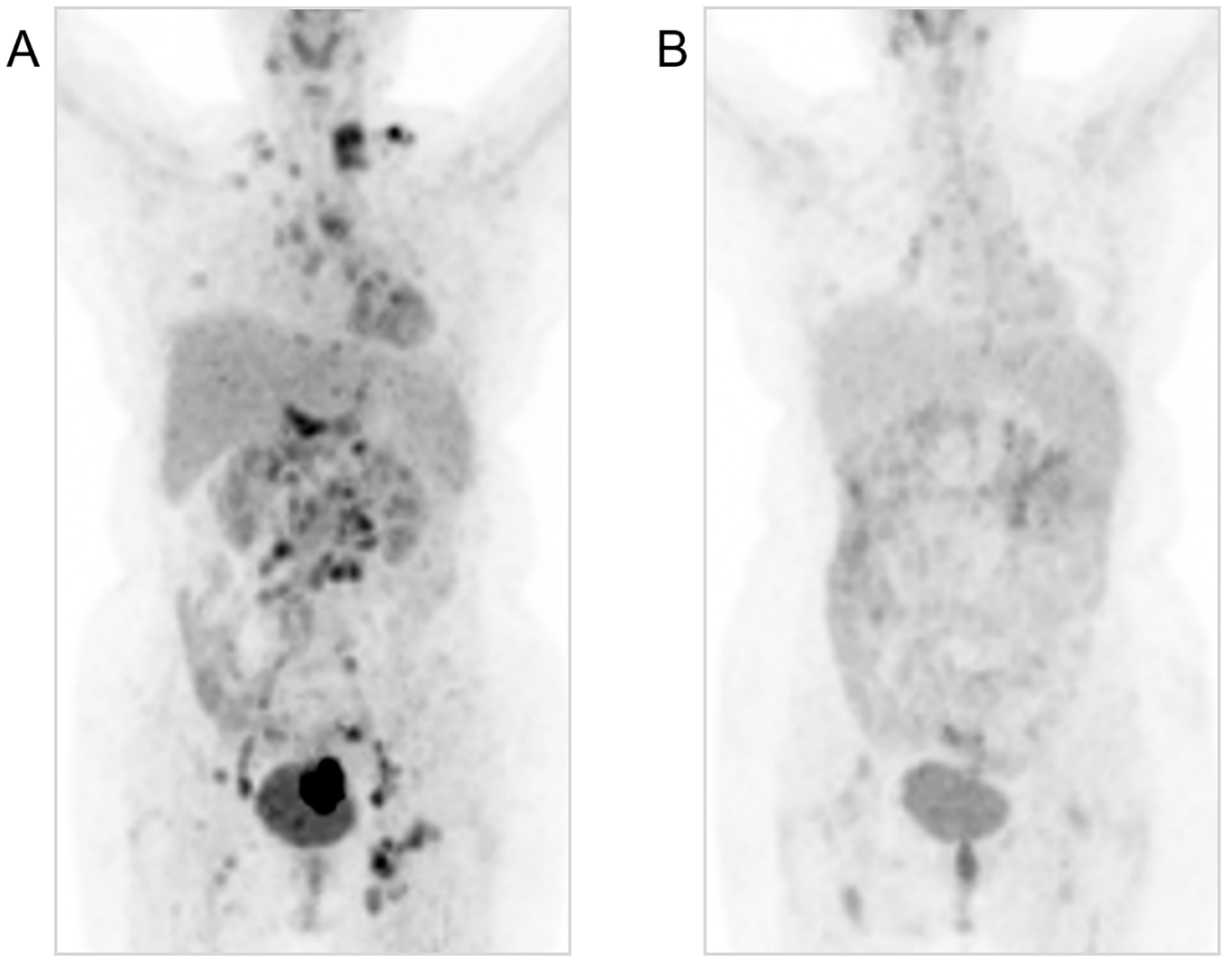
Comparison of PET-CT before traetment **(A)** and after treatment for 1 years **(B)**.

A 55-year-old postmenopausal treatment-naive female was hospitalized for two-month persistent irregular vaginal bleeding. No genetic, family or psychosocial history was reported. A cervical tumor biopsy was performed and examined by two experienced pathologists. Then, she was diagnosed with cervical adenosquamous carcinoma. Immunohistochemistry (IHC) of the cervical tumor showed PD-L1(28-8) (CPS=10) and human epidermal growth factor receptor 2 (HER-2) (3+). Other positive biomarkers included Ki-67 (70%+), P16 (+), CK5/6 (+/-) (**Figure 1A**). The positron emission tomography-computed tomography (PET/CT), which was evaluated by two experience radiologists, revealed a cervical mass with multiple lymph node metastases such as bilateral clavicular region, right internal mammary, mediastinum, left hilus, supradiaphragmatic region, abdominal cavity, retroperitoneum, bilateral pelvic cavity, and bilateral groin. Stage IVB was confirmed according to the 2018 International Federation of Gynecology and Obstetrics (FIGO) Cervical Cancer Staging Guidelines. (**Figure 2A**). To confirm tumor homogeneity, we also conducted a left supraclavicular lymph node biopsy, indicating squamous cell carcinomas (SCC) with PD-L1 (28-8) (CPS=0), and HER-2 (1+). Interestingly, unlike her primary cervical lesions, lymph node metastases did not include any adenocarcinoma component and showed PD-L1 (-) and HER-2 (1+) (**Figure 1B**).

**Figure 3:**
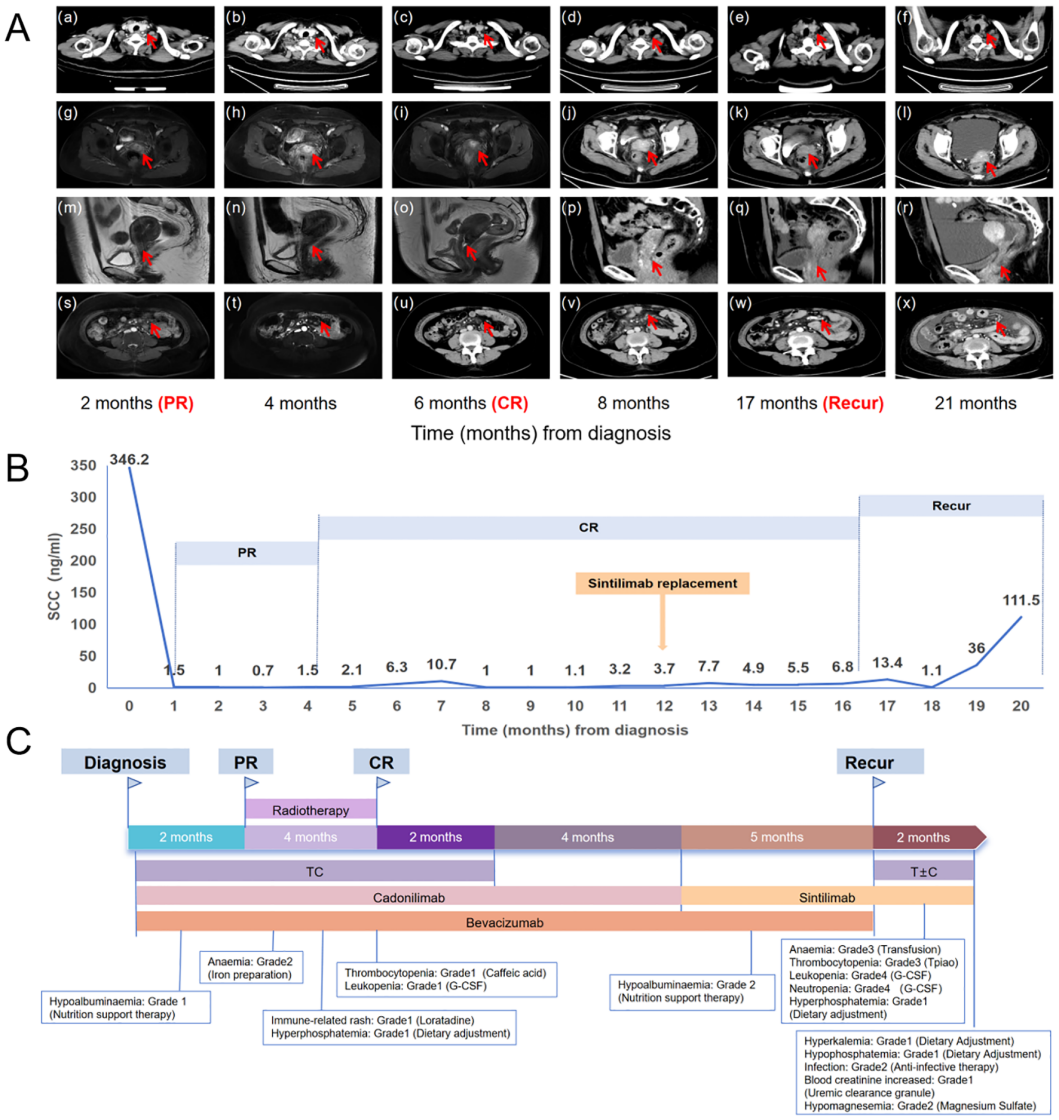
**(A)** The trend of lesion changes during comprehensive treatment. (a-f) The red arrow in the chest CT indicates the left supraclavicular lymph node. (g-i, m-o) The arrow in the MRI indicates the cervical mass. (j-l, p-r) The arrow in the CT indicates the cervical mass. (s, t) The arrow in the MRI indicates the celiac lymph nodes. (u-x) The arrow in the CT indicates the celiac lymph nodes. **(B)** SCC antigen levels during treatment (reference range: 0–1.5 ng/ml). **(C)** The treatment timeline for the patient. T, taxol; C, cisplatin; PR, partial response; CR, complete remission.

Considering that the patient had a cervical tumor with PD-L1 (+) and lymph node metastases with PD-L1(-), cadonilimab was chosen as the best treatment option. Hence, the patient was recommended the following regimen: taxol (175 mg/m²) on day 1, cisplatin (60 mg/m²) on day 1, and cadonilimab (10 mg/kg) on day 2, with an interval of 21 days for six cycles. Then, bevacizumab (15mg/kg) was added at the second cycle. Additionally, local radical radiotherapy was also performed during the treatment. The detailed radiation plan showed below: 1) The 47.25 Gy/27F for volumetric modulated arc therapy (VMAT) in the whole uterus, the vagina, the part of the parametrium, the pelvic lymph node, and the retroperitoneal lymph node; 2) The 45.90 Gy/27F for bilateral inguinal lymphatic area; 3) The 47.60 Gy/28F for additional irradiation of retroperitoneal lymph nodes; 4) Four fractions brachytherapy (27.5165 Gy). The patient’s abdomen magnetic resonance imaging (MRI) and chest computed tomography (CT), which were performed one month after radiotherapy, indicated CR without any signs of tumor. After chemotherapy and radiotherapy were all finished, maintenance therapies with bevacizumab and cadonilimab were continued for five cycles. It is worth mentioning that the patient did not experience any adverse events (AEs) (24) of grade 3-4 during this salvage treatment. The trend of mass change and the fluctuation of SCC are shown in **Figure 2B**, **Figures 3A**, **B**, respectively.

After five cycles of maintenance therapy, cadonilimab was replaced by sintilimab due to patient’s financial constraints. Unfortunately, we observed a recurrence in abdomen CT with large ascites, enlarged abdominal lymph nodes, and thickened peritoneum after 5 cycles of maintenance therapy with sintilimab. Whereupon the patient received taxol + carboplatin (TC) + sintilimab for just one cycle which was suspended by the occurrence of grade four bone marrow suppression and multiple times of infections. The treatment timeline for the patient and AEs were shown in **Figure 3C** and **Table 1**.

**Table 1:** AEs during treatment evaluated by the Common Terminology Criteria for Adverse Events (CTCAE) version 5.0.

	Cadonilimab with other therapy	Sintilimab with other therapy
Relatively severe AEs		
Decreased white blood cell count	Grade 1	Grade 4
Decreased platelet count	Grade 1	Grade 3
Anaemia	Grade 2	Grade 3
Decreased neutrophil count	-	Grade 4
Tolerable AEs		
Immune-related rash	Grade 1	-
Hyperphosphatemia	Grade 1	Grade 1
Hypoalbuminaemia	Grade 1	Grade 2
Hypomagnesemia	-	Grade 2
Infection	-	Grade 2
Hyperkalemia	-	Grade 1
Hypophosphatemia	-	Grade 1
Blood creatinine increased	-	Grade 1

AEs, adverse events.

## Discussion

We reported a R/M CC patient who maintained unexpected CR for 10 months with safety profiles treated with cadonilimab. Surprisingly, metastasis with PD-1 negative also disappeared. As far as we know, this case achieved CR in the shortest cadonilimab treatment period and maintained CR longer than other cases in the literature (25, 26).

In preclinical and clinical studies, the combination of ICIs targeting PD-1 and CTLA-4 has exhibited synergistic antitumor activity (16, 27). In this case, the disseminated CC patients presented CR after being treated with cadonilimab and other combined therapies. However, the patient progressed rapidly after replacing cadonilimab with sintilimab, indicating dual blockade immunotherapy offers substantial advantages over ICI monotherapy (17, 18), which were consistent with the previous studies on the comparison of cadonilimab and pembrolizumab [ORR _cadonilimab_ = 33% (33/100) vs ORR _pembrolizumab_ = 12.2% (12/98)] (28, 29). Such enhanced anti-tumor efficacy of cadonilimab was also observed in lung cancer, rectal cancer, nasopharyngeal cancer, and so on (30–36). The remarkable anticancer mechanism of cadonilimab was worth exploring. Cell binding assays showed that cadonilimab blocked PD-1 binding to PD-L1 and PD-L2 and CTLA-4 binding to B7-1 and B7-2 simultaneously and crosslinked cells expressing PD-1 and CTLA-4. In addition, cadonilimab had a higher affinity for tumor infiltrating lymphocytes in the tumor microenvironment compared with surrounding tissues (23, 37). PD-1 inhibitors and CTLA-4 inhibitors exhibited no cross-resistance and assisted in reshaping immune memory, resulting in a long-term immune response (17).

Moreover, this case might give us two clues for CR possibility after cadonilimab treatment. First, as shown in this case, both the PD-L1-positive cervical primary lesion and the PD-L1-negative left clavicular metastases achieved a CR after treatment, which was consistent with findings of the COMPASSION-13 study (ORR _PD-L1 positive_ = 77.8% (21/27); ORR _PD-L1 negative_ = 70.6% (12/17)), suggesting that cadonilimab could provide clinical benefits for patients with PD-L1-negative CC (30, 38). Second, it was likely that comprehensive treatmentwas the important reason for the remarkable efficacy. Despite patients with PD-L1 CPS < 1 also demonstrated the possibility of an impressive response rate with the treatment of cadonilimab, CR patients still remain a minority. Cadonilimab combined with chemotherapy, radiotherapy, and anti-angiogenic therapy might be an explanation for this CR case. When it came to chemotherapy, many studies showed that chemotherapy not only stimulated tumor antigen release and presentation, resulting in increased activation of tumor-infiltrating lymphocytes, but also synergized with PD-1 pathway blockade to prolong the efficacy of immunotherapy (39, 40). Of note, radiotherapy in the combined treatment of the role also can not be underestimated. Combination treatments with radiotherapy and anti-PD-1 antibodies or anti-CTLA-4 antibodies could activate tumor-specific T cells in the tumor microenvironment (TME), increase the infiltration of CD8-positive T cells, and reduce the accumulation of myeloid-derived suppressor cells (MDSCs) and regulatory T cells, thereby improving anti-tumor immunity (41–44). Besides, the restoration of immune responsiveness induced by anti-PD-1 antibodies or anti-CTLA-4 antibodies and inhibition of angiogenesis after anti-angiogenic therapy could revert the effect resulting in hypoxia on the TME and restore the reciprocal efficacy of the two treatments (45). In conclusion, all these factors might contribute to CR status of the patient.

It is noteworthy that the patient experienced only mild AEs during treatment with cadonilimab, which was in line with COMPASSION-01/03/06/13 and AK104-201, where the incidence of AEs≥grade 3 was only 12% to 26% (30, 31, 35, 46). Although subsequent adverse reactions in the patient might be attributed to toxicity accumulation from previous treatment, the fragment (Fc)-null design of cadonilimab could suggest lower toxicity in this case. The crystallizable fragment (Fc)-null design could eliminate binding to FcγRs and C1q and to minimize lymphocyte loss and antibody-dependent cytokine release from macrophages. What’s more, cadonilimab showed no affinity for FcγRIa, FcγRIIa_H131, FcγRIIIa_V158, FcγRIIIa_F158 or C1q and did not elicit antibody-mediated cell-dependent cytotoxicity, complementary-dependent cell-mediated cytotoxicity or antibody-dependent cellular phagocytosis activity and interleukin-6 (IL-6)/interleukin-8 (IL-8) release. These features all likely contribute to significantly lower toxicities of cadonilimab observed in the clinic (21, 37).

Notably, IHC showed that the cervical tumor was strongly positive for HER-2, indicating HER-2 might play an important role in CR of R/M CC with cadonilimab. Similar findings have been reported in other cancers. Peng J et al (47) reported a patient with HER-2 positive advanced gastroesophageal junction (GEJ) cancer received cadonilimab combined with chemotherapy and achieved CR. The KEYNOTE-811 trial also demonstrated that 15 gastric cancer patients with HER-2 positive obtained CR status by adding pembrolizumab and trastuzumab to chemotherapy (48), which was consistent with our findings. There was the clear evidence of crosstalk between HER-2 and PD-L1 pathways (49, 50). PD-1 inhibitors increased the proportion of intratumoral CD8+ T cells, which could enhance the therapeutic efficacy of HER2-targeted antibody-drug conjugates and reduce tumor growth (51). At the same time, HER2-targeted antibody-drug conjugates could also upregulate PD-L1 expression by upregulating IFN-γlevel (52). In addition, there was a cross-linking between HER-2 positive tumor cells and PD-1 positive T cells in immune synapses. Then, PD-1/HER-2 bispecific antibodies could eliminate tumor cells without antigen recognition (53) Hence, the enhanced antitumor effect of PD-1 inhibitors might be more evident in HER2-overexpressing tumors. However, there is much more to know regarding the underlying interactions pertaining to these two targets. The role of HER-2 in R/M CC patients treated with cadonilimab is worth exploring.

The high expense of specific cancer treatments might influence clinical practice and treatment choices. Although the patient was satisfied with cadonilimab value for its potential for robust and durable responses, she was discouraged by the expense of cadonilimab. To enable the wide usage of immunotherapy, such as cadonilimab and pembrolizumab, much effort and action will needed in order to benefit more patients, such as lower medicine prices and broaden insurance coverage.

There are some limitations that needed to be acknowledged. First, some medical information was incomplete, such as infrequent imaging evaluations and a lack of pathological confirmation for CR status. Second, the safety comparison between cadonilimab and sintilimab might be influenced by several other factors, such as the accumulation of anti-cancer drug toxicity and the side effects of other treatments. Third, more comprehensive and focused studies are required to thoroughly characterize the immunological mechanism of CR status. Overall, we presented a CR case in R/M CC patients treated with cadonilimab, which had excellent performance in anti-cancer effects. This case report will provide valuable information for the future application of cadonilimab in the treatment of R/M CC.

## Conclusion

With the approval of an increasing number of immunotherapeutic agents, the treatment of malignant tumors has entered the era of immunotherapy. ICIs have provided significant benefits for late-stage CC patients, such as increasing the probability of obtaining CR. Cadonilimab offers a promising and effective therapeutic approach for R/M CC. Further investigations are merited to explore its potential mechanisms.

## Data Availability

The original contributions presented in the study are included in the article/supplementary material. Further inquiries can be directed to the corresponding author/s.
